# Effects of MK-886, an inhibitor of the 5-lipoxygenase-activating protein, on peripheral blood mononuclear cells from patients with Chagas' heart disease

**DOI:** 10.1590/0037-8682-0130-2026

**Published:** 2026-09-14

**Authors:** Wanessa Maria dos Santos, Thaís Soares Farnesi de Assunção, João Batista Camargo, Haline Ogata, Maxelle Martins Teixeira, Gabriel Alves Borges dos Santos, Rodrigo Cunha de Sousa, Dalmo Correia, Virmondes Rodrigues, Lucia Helena Faccioli, Alexandre de Paula Rogério

**Affiliations:** 1 Universidade Federal do Triângulo Mineiro, Instituto de Ciências da Saúde, Departamento de Biomedicina, Laboratório de Imunofarmacologia Experimental, Uberaba, MG, Brasil.; 2 Universidade Federal do Triângulo Mineiro, Instituto de Ciências Biológicas e Naturais, Laboratório de Imunologia, Departamento de Microbiologia, Imunologia e Parasitologia, Uberaba, MG, Brasil.; 3 Universidade de São Paulo, Faculdade de Medicina de Ribeirão Preto, Departamento de Bioquímica e Imunologia, Ribeirão Preto, SP, Brasil.; 4 Hospital Pequeno Príncipe, Centro de Pesquisa Clínica do Curitiba, Curitiba, PR, Brasil.; 5 Fundação Getulio Vargas, Escola de Políticas Públicas e Governo, Brasília, DF, Brasil.; 6 Universidade de Uberaba, Uberaba, MG, Brasil.; 7 Universidade Federal do Triângulo Mineiro, Departamento de Clínica Médica, Disciplina de Infectologia, Uberaba, MG, Brasil.; 8 Universidade Federal de Sergipe, Programa de Pós-Graduação em Ciências da Saúde, Aracaju, SE, Brasil.; 9 Universidade de São Paulo, Faculdade de Ciências Farmacêuticas de Ribeirão Preto, Departamento de Análises Clínicas, Toxicológicas e Bromatológicas, São Paulo, SP, Brasil.

**Keywords:** MK-886, Chagas disease, PBMC, *T. cruzi* antigens

## Abstract

**Background:**

Chagas disease, caused by *Trypanosoma cruzi*, remains a major public health challenge in Latin America. Lipid mediators, including leukotrienes, play a key role in modulating the immune response during infection. 5-Lipoxygenase deficiency has been associated with cardioprotective effects in mouse models. Accordingly, this study evaluated the effects of MK-886, a 5-lipoxygenase-activating protein inhibitor, on the immune response of cells from patients with Chagas heart disease.

**Methods:**

Peripheral blood mononuclear cells (PBMCs) from patients with chronic Chagas disease were stimulated with *T. cruzi* antigen in the presence or absence of MK-886. Cytokine production (interferon [IFN]-γ, interleukin [IL]-13, IL-17, IL-10, and tumor necrosis factor [TNF]-α), apoptosis, necrosis, and cell proliferation were assessed.

**Results:**

*T. cruzi* antigen significantly increased cytokine production, cell death, and proliferation in PBMCs from patients with chronic Chagas disease. MK-886 significantly reduced IFN-γ, IL-13, and IL-17 production without altering IL-10 or TNF-α production. Additionally, MK-886 treatment markedly reduced apoptosis, necrosis, and proliferation in *T. cruzi*-stimulated PBMCs from patients with chronic Chagas disease.

**Conclusions:**

MK-886 has the potential to reduce tissue-damaging inflammation and fibrosis associated with Th1, Th2, and Th17 responses without inducing immunosuppression, while IL-10 and TNF-α production remained unchanged in PBMCs from patients with chronic Chagas disease. Accordingly, targeting the leukotriene pathway may delay the progression of cardiac damage in Chagas disease.

## INTRODUCTION

Chagas disease, caused by the parasite *Trypanosoma cruzi*, is a neglected tropical disease with an estimated global prevalence of 275,377 cases in 2019, with most cases occurring in Latin America^1^. The development of distinct clinical forms depends partly on the infecting parasite strain and is also influenced by the host immune response^2^. Chagas disease occurs in two phases: acute and chronic. The acute phase lasts 4‒8 weeks and is asymptomatic in most cases, although up to 30% of patients develop mild flu-like symptoms^3^. After the acute phase, most patients enter the chronic indeterminate phase, which can persist for decades. In some cases, the disease progresses to the chronic cardiac form, which is associated with high morbidity and mortality, and/or the digestive form^4^ ^,^ ^5^.

Lipid mediators, such as leukotrienes (LTs), exert pro-inflammatory effects and may modulate the immune response in Chagas disease^6^ ^,^ ^7^. Leukotriene B₄ (LTB₄) and cysteinyl LTs (LTC_4_, LTD_4_, and LTE_4_) are derived from arachidonic acid through 5-lipoxygenase (5-LO)-mediated oxidation^8^. Pavanelli et al.^9^ reported cardioprotective effects, including attenuated inflammation, reduced collagen deposition, and decreased infiltration of CD4^+^, CD8^+^, and interferon (IFN)-γ-producing cells, but also increased parasite burden in 5-LO-deficient mice experimentally infected with *T. cruzi*. Similarly, 5-LO-deficient mice experimentally infected with *T. cruzi* showed increased plasma IL-6 production^10^. Notably, LTB_4_ inhibited *T. cruzi* replication and induced tumor necrosis factor (TNF)-α production in peritoneal macrophages from wild-type mice. Furthermore, in an experimental model of *T. cruzi* infection, CP-105,696, an LTB_4_ receptor antagonist, markedly increased blood parasitemia compared with vehicle-treated mice. Moreover, TNF-α and IFN-γ production was reduced in CP-105,696-treated *T. cruzi-infected* mice^11^. Thus, the reduction of LTs, particularly LTB_4_, may favor an anti-inflammatory microenvironment that is beneficial for cardiac tissue but may also increase parasitemia, with its detrimental effect likely depending on the parasite burden^9^ ^,^ ^10^. In the present study, we investigated the immunomodulatory effects of MK-886, a 5-LO-activating protein (FLAP) inhibitor, on peripheral blood mononuclear cells (PBMCs) isolated from patients with stage B1 chronic Chagas cardiomyopathy and stimulated with *T. cruzi* antigen.

## METHODS

### Patients

The study protocol was approved by the Human Research Ethics Committee of the Universidade Federal do Triângulo Mineiro (UFTM; approval no. 1943). Patients with chronic Chagas cardiomyopathy were recruited from the Chagas Disease Outpatient Clinic at UFTM, which serves individuals from Uberaba and neighboring municipalities. Eligible participants were classified as stage B1, according to the Latin American Guidelines for the Diagnosis and Treatment of Chagas Cardiomyopathy. This stage is characterized by electrocardiographic abnormalities, including conduction disturbances and/or arrhythmias, without ventricular dysfunction, and mild regional wall-motion abnormalities on echocardiography, with preserved global ventricular systolic function. All participants underwent a standardized clinical evaluation including medical history, physical examination, electrocardiography (ECG), transthoracic echocardiography, chest radiography, contrast esophagography, and barium enema. Infection with *T. cruzi* was confirmed by at least two positive serological assays, including indirect immunofluorescence, indirect hemagglutination, and enzyme-linked immunosorbent assay (ELISA). Participants were eligible if they were 18-60 years old, had not received benznidazole treatment within the previous five years, and provided written informed consent. Exclusion criteria included stage B2, C, or D cardiac involvement; digestive manifestations of Chagas disease, including megaesophagus or megacolon; hypertension, diabetes mellitus, renal, hepatic, or thyroid disease; and the use of a permanent cardiac pacemaker. 

The sample consisted of 5 patients with chronic Chagas disease (80% female and 20% male), with a mean age of 64.00 ± 13.98 years (mean ± standard deviation; range, 39-71 years). The median time since diagnosis was 20 years. Two patients (40%) had at least one comorbidity (osteoporosis, n = 1 [20%]; gastric ulcer, n = 1 [20%]). Of the 10 patients invited to participate, five (50%) were enrolled, whereas the remaining five were excluded because of thyroid disorders. 

### Trypanosoma cruzi antigen preparation

Epimastigotes of the Y strain of *T. cruzi* were cultured in Schneider's insect medium supplemented with 50 mM L-glutamine, 40 µg/mL gentamicin, and 20% fetal calf serum until the stationary phase was reached. Parasites were harvested by centrifugation, washed three times with phosphate-buffered saline (PBS), and adjusted to a final concentration of 2 × 10^8^ epimastigotes/mL in sterile distilled water. Cell disruption was achieved by five consecutive freeze-thaw cycles. The lysates were subsequently centrifuged at 5,000 × *g* for 30 min at 4 °C to remove cellular debris, and the resulting supernatants were collected as crude *T. cruzi* antigen preparations. Total protein concentration was determined using the Bradford protein assay before the *in vitro* experiments.

### PBMC stimulation and treatment

Freshly isolated PBMCs (2 × 10^6^ cells/mL) were distributed into 96-well plates and treated with MK-886 (1 μM)^12^ or vehicle (ethanol; final concentration 0.04%)^13^ 30 min before stimulation with *T. cruzi* antigen (5 µg/mL) and incubated at 37 ºC for 48 or 120 h. Cytokine analysis was performed at different time points (48 and 120 h) based on previous experience and published studies from our group^14^ ^,^ ^15^. 

### Cytokine quantification

Cell-free supernatants were harvested at 48 h for the determination of TNF-α and IL-10, and at 120 h for the measurement of IFN-γ, IL-13, and interleukin (IL)-17 following stimulation with *T. cruzi* antigen. Cytokine concentrations were determined using commercially available ELISA kits (R&D Systems or BD Pharmingen), according to the manufacturers' instructions. Cytokine levels were calculated from standard curves and expressed as pg/mL.

### Carboxyfluorescein succinimidyl ester (CFSE)-based PBMC proliferation analysis

PBMCs were seeded at a density of 2 × 10^6^ cells/mL in 96-well culture plates and labeled with 10 μM CFSE before stimulation. Following 48 h of stimulation with *T. cruzi* antigen, non-adherent cells were collected, washed, and resuspended for flow cytometric analysis. Cell viability was assessed by the Trypan blue exclusion method prior to acquisition. CFSE fluorescence was evaluated in 10,000 viable events using a FACSCalibur flow cytometer (BD Biosciences), and data were acquired using CellQuest software (BD Biosciences). Flow cytometry data were subsequently analyzed using FlowJo software (version 10).

### Assessment of PBMC viability and apoptosis

PBMCs (2 × 10^6^ cells/mL) were stained with fluorescein isothiocyanate (FITC)-conjugated annexin V and propidium iodide (PI) using the BD Annexin V: FITC Apoptosis Detection Kit I (BD Biosciences, Cat. No. 556547), according to the manufacturer's instructions. Briefly, cells from each experimental group were harvested by centrifugation (300 × *g*, 5 min), washed twice with ice-cold PBS, and resuspended in binding buffer. The cell suspensions were incubated with annexin V-FITC and PI for 15 min at room temperature in the dark. Samples were acquired using a BD FACSCalibur flow cytometer (BD Biosciences). An initial gate based on forward scatter (FSC) and side scatter (SSC) parameters was applied to exclude cellular debris, followed by doublet discrimination using FSC-A versus FSC-H to identify singlet cells. Cell viability, apoptosis, and necrosis were subsequently evaluated within the singlet population based on annexin V-FITC and PI staining patterns. Cells were classified as viable (annexin V−/PI−), early apoptotic (annexin V+/PI−), late apoptotic or secondary necrotic (annexin V+/PI+), or primary necrotic (annexin V−/PI+). Cell viability was expressed as the percentage of viable cells relative to the total singlet population. Data were acquired using CellQuest software (BD Biosciences), and flow cytometry data were analyzed with FlowJo software (version 10).

### Statistical analysis

Data are expressed as the mean ± standard error of the mean (SEM). Statistical differences between groups were assessed using one-way analysis of variance (ANOVA) followed by Tukey's multiple-comparisons post hoc test. The corresponding F statistics and *p* values are reported where appropriate. Statistical significance was defined as *p* < 0.05.

## RESULTS

### MK-886 reduces IFN-γ, IL-17, and IL-13 production in PBMCs from patients with chronic Chagas disease

**FIGURE 1:**
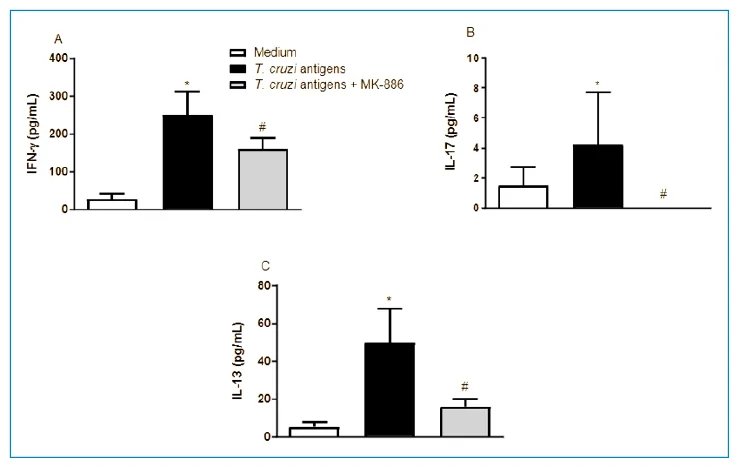
Effect of MK-886 on IFN-γ, IL-17, and IL-13 production by PBMCs from patients with chronic Chagas cardiomyopathy. Peripheral blood mononuclear cells (PBMCs) were cultured with *Trypanosoma cruzi* antigen (5 μg/mL) in the presence or absence of MK-886 (1 μM). After 120 h of stimulation, culture supernatants were collected, and IFN-γ **(A)**, IL-17 **(B)**, and IL-13 **(C)** concentrations were determined by ELISA. Data are expressed as the mean ± SEM (n = 5). p < 0.05 versus the control group; #p < 0.05 versus the T. cruzi antigen-stimulated group.

*T. cruzi* antigen stimulation increased IFN-γ, IL-17, and IL-13 concentrations in PBMCs from patients with chronic Chagas disease compared with unstimulated PBMCs (Figure 1A‒C ). MK-886 significantly reduced IFN-γ (*F*(2, 12) = 19.98; *p* < 0.0001; Figure 1A), IL-17 (*F*(2, 12) = 3.748; *p* = 0.0479; Figure 1B), and IL-13 (*F*(2, 12) = 10.61; *p* = 0.0013; Figure 1C) concentrations in *T. cruzi* antigen-stimulated PBMCs compared with PBMCs stimulated with *T. cruzi* antigen alone. 

**SUPPLEMENTARY FIGURE 1:**
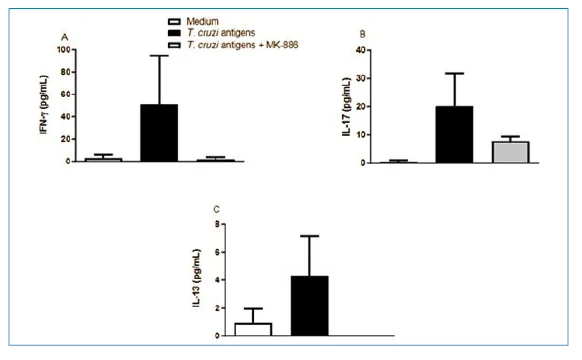
MK-886 did not reduce IFN-γ, IL-17, and IL-13 production in PBMCs from healthy individuals stimulated with *T. cruzi* antigen. Peripheral blood mononuclear cells (PBMCs) were stimulated by *Trypanosoma cruzi* antigen (5 µg/mL) in the presence or absence of MK-886 (1 µM). After 120 h of stimulation with *T. cruzi* antigen, culture supernatants were collected, and IFN-γ **(A)**, IL-17 **(B)**, and IL-13 (C) concentrations were determined by ELISA. Data are presented as the mean ± SEM (n = 4).

In PBMCs from healthy individuals, *T. cruzi* antigen stimulation tended to increase IFN-γ, IL-17, and IL-13 concentrations, but the differences were not statistically significant compared with unstimulated PBMCs ( Supplementary Figures 1 A‒C ). MK-886 treatment did not significantly reduce IFN-γ (*F*(2, 9) = 1.242; *p* = 0.3339; Supplementary Figure 1A ), IL-17 (*F*(2, 9) = 2.149; *p* = 0.1726, Supplementary Figure 1B ), and IL-13 (*F*(2, 9) = 1.696; *p* = 0.2371; Supplementary Figure 1C ) concentrations in *T. cruzi* antigen-stimulated PBMCs compared with PBMCs stimulated with *T. cruzi* antigen alone. 

No changes in cytokine production were observed following vehicle treatment in either unstimulated or *T. cruzi* antigen-stimulated cells (data not shown).

### MK-886 does not alter IL-10 and TNF-α production by PBMCs from patients with chronic Chagas disease

**FIGURE 2:**
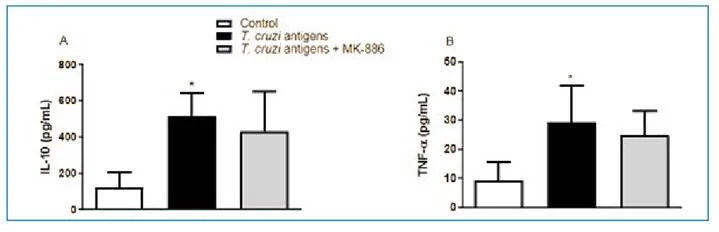
Effect of MK-886 on IL-10 and TNF-α production by PBMCs from patients with chronic Chagas cardiomyopathy. Peripheral blood mononuclear cells (PBMCs) were incubated with *Trypanosoma cruzi* antigen (5 μg/mL) in the presence or absence of MK-886 (1 μM). After 48 h, culture supernatants were harvested, and IL-10 **(A)** and TNF-α **(B)** concentrations were quantified by ELISA. Data are presented as the mean ± SEM (n = 5). *p* < 0.05 versus the control group.

**SUPPLEMENTARY FIGURE 2:**
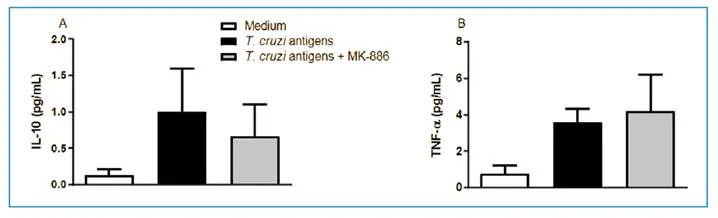
MK-886 did not alter IL-10 and TNF-σ production in PBMCs from healthy individuals stimulated with *T. cruzi* antigen. Peripheral blood mononuclear cells (PBMCs) were stimulated by *Trypanosoma cruzi* antigen (5 µg/mL) in the presence or absence of MK-886 (1 µM). After 48 h of stimulation with *T. cruzi* antigen, culture supernatants were collected, and IL-10 **(A)** and TNF-σ **(B)** concentrations were determined by ELISA. Data are presented as the mean ± SEM (n = 4). *p < 0.05 versus the control group and #p < 0.05 versus the *T. cruzi* antigen group.

*T. cruzi* antigen stimulation increased IL-10 and TNF-α concentrations in PBMCs from patients with chronic Chagas disease compared with unstimulated PBMCs (Figures 2A, B ). MK-886 did not significantly alter IL-10 (*F*(2, 8) = 1.150; *p* = 0.3639; Figure 2A) and TNF-α (*F*(2, 9) = 3.318; *p* = 0.0833; Figure 2B) concentrations in *T. cruzi* antigen-stimulated PBMCs compared with PBMCs stimulated with *T. cruzi* antigen alone. In PBMCs from healthy individuals, *T. cruzi* antigen stimulation increased IL-10 and TNF-α concentrations, but the differences were not statistically significant compared with unstimulated PBMCs (Supplementary Figure 2A‒B ). MK-886 did not significantly alter IL-10 (*F*(2, 9) = 1.051; *p* = 0.3888,  Supplementary Figure 2A) and TNF-α (*F*(2, 9) = 2.083; *p* = 0.1806;  Supplementary Figure 2B) concentrations in *T. cruzi* antigen-stimulated PBMCs compared with PBMCs stimulated with *T. cruzi* antigen alone. No alteration in cytokine production was observed following vehicle treatment in either unstimulated or *T. cruzi* antigen-stimulated cells (data not shown). Based on these findings, subsequent experiments were conducted using only PBMCs from patients with chronic Chagas disease.

### MK-886 attenuates PBMC proliferation

**FIGURE 3:**
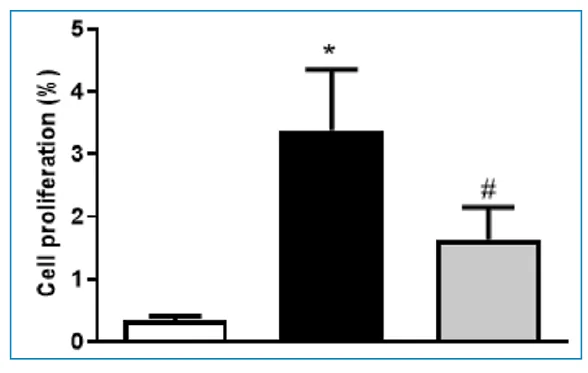
Effect of MK-886 on the proliferative response of PBMCs from patients with chronic Chagas cardiomyopathy. Peripheral blood mononuclear cells (PBMCs) were isolated, labeled with CFSE, and cultured with *Trypanosoma cruzi* antigen (5 μg/mL) in the presence or absence of MK-886 (1 μM). Cells were collected after 48 h and analyzed by flow cytometry. Data are presented as the mean ± SEM (n = 4). *p* < 0.05 versus the control group; #*p* < 0.05 versus the *T. cruzi* antigen-stimulated group.

**SUPPLEMENTARY FIGURE 3:**
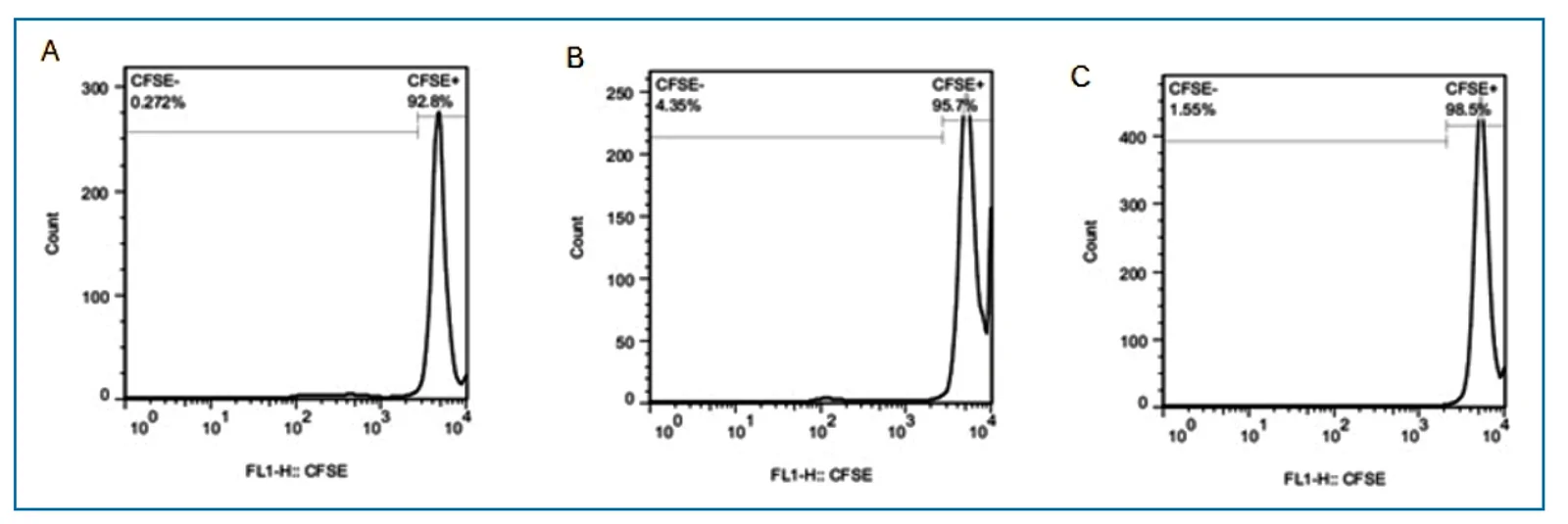
Representative CFSE histogram showing PBMC proliferation. Carboxyfluorescein succinimidyl ester (CFSE)-labeled peripheral blood mononuclear cells (PBMCs) were cultured either unstimulated **(A),** stimulated with *Trypanosoma cruzi* antigen **(B),** or stimulated with *T. cruzi antigen* in the presence of MK-886 **(C)** for 48 h. Cells were analyzed by flow cytometry. The x-axis represents CFSE fluorescence intensity (log scale), and the y-axis indicates cell count.

*T. cruzi* antigen stimulation significantly enhanced PBMC proliferation in cells isolated from patients with chronic Chagas cardiomyopathy compared with unstimulated cultures. Treatment with MK-886 markedly reduced PBMC proliferation compared with PBMCs stimulated with *T. cruzi* antigen alone (*F*(2,9) = 6.937, *p* = 0.0275; Figure 3 and Supplementary Figure 3). Vehicle treatment did not alter PBMC proliferation in either unstimulated or *T. cruzi* antigen-stimulated cells (data not shown).

### MK-886 reduces the number of apoptotic and necrotic PBMCs

**FIGURE 4:**
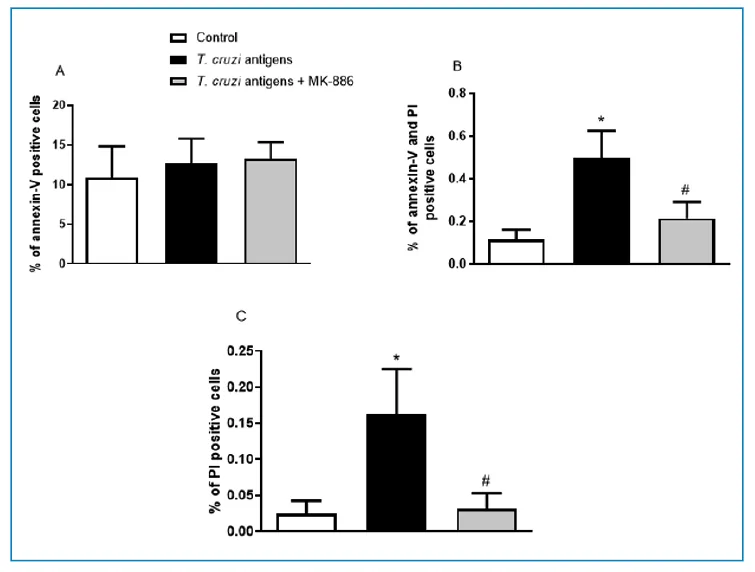
Effect of MK-886 on apoptosis and necrosis in PBMCs from patients with chronic Chagas cardiomyopathy. Peripheral blood mononuclear cells (PBMCs) were stimulated with *Trypanosoma cruzi* antigen (5 μg/mL) in the presence or absence of MK-886 (1 μM). Apoptosis and necrosis were evaluated by Annexin V/PI staining, allowing the discrimination of early apoptotic (Annexin V⁺/PI⁻) **(A)**, late apoptotic/secondary necrotic (Annexin V⁺/PI⁺) **(B)**, and necrotic (Annexin V⁻/PI⁺) **(C)** cells. Data are presented as the mean ± SEM (n = 5). *p* < 0.05 versus the control group; #*p* < 0.05 versus the *T. cruzi* antigen-stimulated group.

No differences were observed in the percentage of early apoptosis (annexin V+/PI−) between the groups (*F*(2, 12) = 0.1568; *p* = 0.8566; Figure 4A). *T. cruzi* antigen stimulation significantly increased the percentage of late apoptotic/secondary necrotic (annexin V+/PI+) (Figure 4B) and necrotic (annexin V−/PI+) cells (Figure 4C) compared with the control group. Furthermore, MK-866 treatment significantly reduced late apoptotic/secondary necrotic cells (*F*(2, 12) = 5.001; *p* = 0.0263; Figure 4B) and necrotic cells (*F*(2, 12) = 5.615; *p* = 0.0190; Figure 4C) compared with PBMCs stimulated with *T. cruzi* antigen alone. No changes in the percentage of apoptotic and necrotic cells were observed following vehicle treatment in either unstimulated or *T. cruzi* antigen-stimulated cells (data not shown).

## DISCUSSION

The pathogenesis of Chagas disease involves complex interactions between *T. cruzi* and the host immune system, particularly with macrophages. Chronic infection progresses to cardiomyopathy in approximately 30% of cases^16^. Parasite persistence and host immune responses are closely linked in chronic myocardial damage^17^. Pieralisi et al.^18^ investigated the cytokine profiles of PBMCs from patients with Chagas disease, revealing significant differences in inflammatory marker expression compared with healthy individuals, which were associated with disease manifestations. De Melo et al.^19^ provided foundational insights into the role of macrophages during *T. cruzi* infection, highlighting the crucial role of monocyte-derived macrophage activation in parasite clearance from cardiac tissue. 

LTs are important lipid mediators involved in the regulation of inflammatory and infectious responses, contributing to processes such as increased vascular permeability, edema formation, leukocyte chemotaxis, lysosomal enzyme release, neutrophil degranulation, adhesion molecule expression, defensin production, and phagocytosis^20^ ^-^ ^23^. Pharmacological inhibition of LT signaling has shown beneficial effects in several cardiovascular disorders^24^ ^-^ ^26^. Consistent with these findings, evidence supports a role for LTs in the pathogenesis of Chagas cardiomyopathy through modulation of the host immune response. Moreover, *in vitro* studies have shown that pretreatment with the lipoxygenase inhibitor nordihydroguaiaretic acid (NDGA) or the cysteinyl LT receptor antagonist FPL 55712 attenuated the positive inotropic and chronotropic responses induced by T lymphocytes from patients with chronic Chagas cardiomyopathy and *T. cruzi*-infected mice^27^ ^,^ ^28^. In addition, LTs enhance the phagocytic and trypanocidal activities of murine macrophages exposed to *T. cruzi* trypomastigotes, supporting their role in parasite control^6^ ^,^ ^11^. Conversely, pharmacological inhibition or genetic disruption of LT biosynthesis reduces IFN-γ-mediated trypanocidal activity in murine macrophages, increases parasitemia during experimental *T. cruzi* infection, and decreases inflammatory cell infiltration into cardiac tissue^6^ ^,^ ^9^ ^-^ ^11^ ^,^ ^29^. Reduced LT biosynthesis is also associated with fewer infiltrating CD4^+^, CD8^+^, and IFN-γ-producing cells, reduced cardiac fibrosis, decreased TNF-α and IFN-γ production, and increased IL-10 levels in the heart. Collectively, these findings indicate that LTs play a central role in regulating both antiparasitic immunity and the inflammatory processes that contribute to the development and progression of chronic Chagas cardiomyopathy. 

Pereira et al.^30^ provided crucial evidence of immune dysregulation in chronic Chagas cardiomyopathy, characterized by a predominant Th1-type immune response and increased production of pro-inflammatory cytokines, particularly IFN-γ and TNF-α, by mononuclear cells infiltrating the myocardium. These mediators promote T-cell recruitment and activation, contributing to the persistence, severity, and progression of myocardial inflammation^31^ ^,^ ^32^. Similarly, Th17 responses play a pivotal role in promoting inflammation and recruiting neutrophils during *T. cruzi* infection, contributing to chronic inflammation, particularly in the heart, and the development of Chagas cardiomyopathy^33^. In an experimental model of *Toxoplasma gondii*, MK-886 reduced IFN-γ production in the ileum^34^. MK-886 also reduced IL-17-induced neutrophil migration following intra-articular injection in mice^35^. Conversely, in animals immunized using a heterologous BCG/DNA-HSP65 prime-boost strategy and subsequently infected with *Mycobacterium tuberculosis*, MK-886 increased IL-17 production in the lungs. In the present study, MK-886 reduced IFN-γ and IL-17 production in *T. cruzi* antigen-stimulated PBMCs from patients with chronic Chagas cardiomyopathy. Our results suggest that the beneficial effects of LT inhibition in attenuating chronic Chagas myocarditis may be attributed, at least in part, to an improved balance between Th1 and Th17 responses. Although the interplay between pro- and anti-inflammatory cytokines plays a crucial role in determining the clinical outcomes of Chagas disease, and anti-TNF-α therapy increases the frequency of IL-10-producing splenic CD4^+^ T-cells^36^, our results showed that MK-886 did not significantly alter TNF-α or IL-10 production in *T. cruzi* antigen-stimulated PBMCs from patients with chronic Chagas cardiomyopathy. These findings suggest that the immunomodulatory effects of MK-886 may occur independently of TNF-α and IL-10 regulation. 

IL-13, a cytokine predominantly produced by Th2 lymphocytes, has been associated with increased susceptibility to *T. cruzi* infection, largely because of its ability to suppress protective Th1-mediated immune responses^37^. Beyond its immunomodulatory effects, IL-13 also plays an important role in extracellular matrix remodeling and tissue fibrosis by promoting transforming growth factor-β (TGF-β) production by fibroblasts^38^. In the present study, MK-886 markedly reduced IL-13 production in *T. cruzi* antigen-stimulated PBMCs from patients with chronic Chagas cardiomyopathy. These findings suggest that FLAP inhibition may help attenuate the profibrotic environment associated with chronic Chagas myocarditis, potentially limiting the progression of cardiac remodeling and fibrosis. Myocardial fibrosis is a hallmark of Chagas cardiomyopathy and is present even in early, asymptomatic stages such as stage B1. Cardiac fibrosis progresses continuously at an estimated rate of roughly 7-8% per year^39^. The median time since diagnosis among the patients included in this study was 20 years. Therefore, the reduction in profibrotic cytokine production may help limit excessive collagen deposition and cardiac fibrosis. Apoptosis is a physiological process that normally does not elicit an inflammatory immune response. During pathological conditions, however, increased apoptosis and impaired clearance of apoptotic cells may contribute to inflammation through the release of pro-inflammatory signals. 

Patients with Chagas cardiomyopathy exhibit increased apoptosis in response to *T. cruzi* antigen exposure compared with those with the indeterminate form of the disease^40^. Apoptosis of T lymphocytes and macrophages may help regulate persistent inflammation and limit cardiac tissue damage in chronic Chagas disease^41^. Notably, the effects of MK-886 on apoptosis appear to be context dependent. While MK-886 has been reported to induce apoptosis in human malignant glioma cells^42^ ^,^ ^43^, it did not induce apoptosis in breast cancer cells^44^. Additionally, MK-886 inhibited apoptotic, but not necrotic, cell death^45^. Our results demonstrated that MK-886 reduced the percentage of late apoptotic cells and necrotic PBMCs from patients with chronic Chagas cardiomyopathy following stimulation with *T. cruzi* antigen. This effect may help preserve the immune cell repertoire while limiting the release of damage-associated molecular patterns, thereby attenuating inflammatory signaling within the cardiac microenvironment. Our findings demonstrated that *T. cruzi* antigen stimulation markedly enhanced PBMC proliferation in patients with chronic Chagas cardiomyopathy compared with unstimulated cultures, corroborating previous observations reported by our group^13^ and others^46^ ^-^ ^48^. Although MK-886 has also been shown to inhibit cell proliferation in gastric cancer cells^49^, these findings should be interpreted with caution, as malignant cells exhibit profound alterations in cell-cycle regulation and proliferative signaling that differ substantially from those of normal immune cells. In the present study, MK-886 markedly reduced the proliferative response of antigen-stimulated PBMCs from patients with chronic Chagas cardiomyopathy. This effect may limit excessive T-cell activation and expansion, thereby attenuating the inflammatory response, as supported by the concomitant reduction in IFN-γ and IL-17 production. Given the central role of activated lymphocytes in the pathogenesis of chronic Chagas myocarditis, the ability of MK-886 to restrain PBMC proliferation suggests a potential immunomodulatory effect that may help reduce the persistent inflammatory environment associated with cardiac tissue injury and disease progression.

The differential modulation of Th1, Th2, and Th17-associated cytokines, but not IL-10 or TNF-α, following MK-886-induced LT inhibition may reflect differences in the underlying inflammatory pathways. IFN-γ production is regulated by T-bet, STAT4, and NFAT^50^. Reduced LT biosynthesis is associated with impaired IFN-γ production^51^. CCAAT/enhancer-binding proteins (C/EBPs) are essential downstream mediators of the IL-17 signaling pathway^52^, and their expression is reduced in the absence of LTs^53^. Similarly, cysteinyl leukotrienes provide a critical signal for Th2-cell activation through the NFAT signaling pathway^54^, and the absence of LT-mediated activation impairs the intracellular signaling required for Th2 responses^55^. TNF-α release is predominantly driven by inflammatory stimuli, including MyD88-dependent Toll-like receptor signaling and NF-κB activation, pathways that may operate independently of 5-LO-derived mediators^56^ ^,^ ^57^. Similarly, IL-10 expression is regulated by transcription factors, including c-Maf and members of the STAT pathways. Accordingly, LT inhibitors may restore or increase IL-10 production^58^. Further investigation of these signaling pathways is needed to clarify the mechanisms by which MK-886 modulates immune responses in patients with Chagas cardiomyopathy. Overall, the available evidence suggests that LTs function as a “molecular switch” balancing inflammatory responses and parasite control in Chagas disease. Although LT inhibition may help reduce the inflammatory microenvironment in the heart, it may also impair trypanocidal activity. However, our study demonstrated a potentially important mechanism of action of MK-886 in chronic Chagas cardiomyopathy, as it promoted anti-inflammatory effects without inducing broad immunosuppression, evidenced by the absence of changes in IL-10 and TNF-α production. This profile may preserve immune regulatory mechanisms involved in parasite control. Further *in vitro*, *ex vivo*, and *in vivo* studies are needed to confirm these findings and elucidate the underlying mechanisms. 

Our study has several limitations. The use of PBMCs may not fully reflect the phenotype, gene expression, and function of immune cells actively residing within specific tissues. Moreover, PBMCs are a heterogeneous cell population, and their composition may vary according to donor age, physiological status, and sample processing time. In addition, the relatively small number of patients included in this study represents an important limitation. Furthermore, proliferation, apoptosis/necrosis, and/or cytokine production should be evaluated in specific immune cell subsets, including CD4^+^ T (helper) cells, CD8^+^ T (cytotoxic) cells, and dendritic cells. Finally, we did not investigate the intracellular signaling pathways underlying MK-886-mediated modulation of cytokine production. Elucidating these molecular mechanisms will be important for understanding the immunomodulatory effects of FLAP inhibition. Further experimental and clinical studies are needed to determine whether targeting the LT pathway is a safe and effective therapeutic strategy for patients with chronic Chagas cardiomyopathy, with the potential to attenuate the persistent inflammatory response that contributes to cardiac injury and disease progression. In summary, our findings demonstrate that MK-886 selectively modulates *T. cruzi* antigen-specific responses in PBMCs from patients with stage B1 Chagas cardiomyopathy by reducing Th1-, Th2-, and Th17-associated cytokine production and PBMC proliferation without altering TNF-α and IL-10 production. Although these anti-inflammatory effects support further investigation of the underlying molecular mechanisms and the potential clinical applicability of FLAP inhibition, the possibility of impaired pathogen control should be carefully considered.

## Data Availability

Research data is only available upon request.

## References

[1] Gómez-Ochoa SA, Rojas LZ, Echeverría LE, Muka T, Franco OH (2022). Global, regional, and national trends of Chagas disease from 1990 to 2019: comprehensive analysis of the Global Burden of Disease Study. Glob Heart.

[2] Vieira PM, Francisco AF, Machado EM, Nogueira NC, Fonseca KS, Reiset AB (2012). Different infective forms trigger distinct immune response in experimental Chagas disease. PLoS One.

[3] Couceiro KDN, Ortiz JV, Silva EHS, Sousa DRT, Andrade RC, Brandão ARJ (2022). Myocardial injury in patients with acute and subacute Chagas disease in the Brazilian Amazon using cardiovascular magnetic resonance. J Am Heart Assoc.

[4] Li SX, Soles EO, Sharma PS, Rao AK (2023). Diagnosis of chronic Chagas cardiomyopathy using a multimodality imaging approach. Eur Heart J Case Rep.

[5] Rassi A, Marin-Neto JA, Rassi A (2017). Chronic Chagas cardiomyopathy: pathogenic mechanisms and the efficacy of etiological treatment following the BENEFIT trial. Mem Inst Oswaldo Cruz.

[6] Rogerio AP, Anibal FF (2012). Role of leukotrienes on protozoan and helminth infections. Mediators Inflamm.

[7] Molina-Berríos A, Campos-Estrada C, Henriquez N, Faúndez M, Torres G, Castillo C (2013). Protective role of acetylsalicylic acid in experimental Trypanosoma cruzi infection: evidence of a 15-epi-lipoxin A4-mediated effect. PLoS Negl Trop Dis.

[8] Butola LK, Dhok A, Ambad R, Kanyal D, Jha RK (2021). Leukotrienes and inflammation: a review. Indian J Forensic Med Toxicol.

[9] Pavanelli WR, Gutierrez FRS, Mariano FS, Prado CM, Ferreira BR, Teixeira MM (2010). 5-Lipoxygenase is a key determinant of acute myocardial inflammation and mortality during Trypanosoma cruzi infection. Microbes Infect.

[10] Panis C, Mazzuco TL, Costa CZ, Victorino VJ, Tatakihara VLH, Yamauchi LM (2011). Trypanosoma cruzi: effect of the absence of 5-LO-derived leukotrienes on cytokines and nitric oxide in cardiac tissue during acute infection. Exp Parasitol.

[11] Talvani A, Machado FS, Santana GC, Klein A, Barcelos L, Silva JS (2002). Leukotriene B4 induces nitric oxide synthesis in T cruzi-infected macrophages and mediates resistance to infection. Infect Immun.

[12] Oliveira SH, Canetti C, Ribeiro RA, Cunha FQ (2008). Neutrophil migration induced by IL-1beta depends on LTB4 from macrophages and TNF-alpha/IL-1beta from mast cells. Inflammation.

[13] Costa-Junior HM, Mendes AN, Davis GHNG, Cruz CM, Ventura ALM, Serezaniet CH (2009). ATP-induced apoptosis involves a Ca2+-independent phospholipase A2 and 5-lipoxygenase in macrophages. Prostaglandins Other Lipid Mediat..

[14] Ogata H, Teixeira MM, Sousa RC, Silva MV, C D, R V (2016). Effects of aspirin-triggered resolvin D1 on PBMC from patients with Chagas heart disease. Eur J Pharmacol.

[15] Crema E, Monteiro IO, Gomes MGZ, Silva AA, Rodrigues V (2006). Evaluation of cytokines (MIG, IFN-gamma, TNF-alpha, IL-4, IL-5, and IL-10) during the different evolutive phases of chagasic esophagopathy. Clin Immunol.

[16] Alba-Alvarado MC, Cabrera-Bravo M, Zenteno E, Salazar-Schetino PM, Bucio-Torres MI (2024). The functions of cytokines in the cardiac immunopathogenesis of Chagas disease. Pathogens.

[17] Marin-Neto JA, Cunha-Neto E, Maciel BC, Simões MV (2007). Pathogenesis of chronic Chagas heart disease. Circulation.

[18] Pieralisi AV, Cevey ÁC, Penas FN, Prado N, Mori A, Giliet M (2022). Fenofibrate increases non-classical monocytes and modulates cytokines in asymptomatic Chagas patients. Front Cell Infect Microbiol.

[19] Melo AS, Lorena VM, Moura Braz SC, Docena C, Miranda Gomes Y (2012). IL-10 and IFN-gamma gene expression in chronic Chagas patients after stimulation with recombinant antigens. Cytokine.

[20] Peters-Golden M, Henderson WR (2007). Leukotrienes. N Engl J Med.

[21] Machado FS, Mukherjee S, Weiss LM, Tanowitz HB, Ashton AW (2011). Bioactive lipids in Trypanosoma cruzi infection. Adv Parasitol.

[22] Lewis RA, Austen KF, Soberman RJ (1990). Leukotrienes and other 5-lipoxygenase pathway products: biochemistry and pathobiology. N Engl J Med.

[23] Flamand L, Tremblay MJ, Borgeat P (2007). Leukotriene B4 triggers the in vitro and in vivo release of potent antimicrobial agents. J Immunol.

[24] Hoxha M, Rovati GE, Cavanillas AB (2017). The leukotriene receptor antagonist montelukast and its possible role in the cardiovascular field. Eur J Clin Pharmacol.

[25] Wang X, Baskaran L, Chan M, Boisvert W, Hausenloy DJ (2023). Targeting leukotriene biosynthesis to prevent atherosclerotic cardiovascular disease. Cond Med.

[26] Werz O, Steinhilber D (2006). Therapeutic options for 5-lipoxygenase inhibitors. Pharmacol Ther.

[27] Gorelik G, Borda E, Postan M, Gonzalez Cappa S, Sterin-Borda L (1992). T lymphocytes from T cruzi-infected mice alter heart contractility: participation of arachidonic acid metabolites. J Mol Cell Cardiol.

[28] Bracco MME, Sterin-Borda L, Fink S (1984). Stimulatory effect of lymphocytes from Chagas' patients on spontaneously beating rat atria. Clin Exp Immunol.

[29] Borges CL, Cecchini R, Tatakihara VLH, Malvezi AD, Yamada-Ogatta SF, Rizzo LV (2009). 5-Lipoxygenase plays a role in parasite burden control and contributes to oxidative damage of erythrocytes in murine Chagas disease. Immunol Lett..

[30] Pereira IR, Vilar-Pereira G, Silva AA, Moreira OC, Britto C, Sarmento EDM (2014). Tumor necrosis factor is a therapeutic target for immunological imbalance and cardiac abnormalities in chronic experimental Chagas heart disease. Mediators Inflamm.

[31] Nogueira LG, Santos RH, Ianni BM, Fiorelli AI, Mairena EC, Benvenuti LA (2012). Myocardial chemokine expression and myocarditis severity in Chagas cardiomyopathy are controlled by CXCL9 and CXCL10 polymorphisms. PLoS Negl Trop Dis.

[32] Cunha-Neto E, Chevillard C (2014). Chagas disease cardiomyopathy: immunopathology and genetics. Mediators Inflamm.

[33] Strauss M, Palma-Vega M, Casares-Marfil D (2020). Genetic polymorphisms of IL17A associated with Chagas disease: meta-analysis in Latin American populations. Sci Rep.

[34] Araujo ECB, Briceño MPP, Cariaco Y, Oliveira MC, Almeida MPO, Miranda NC (2022). Toxoplasma gondii infection decreases intestinal 5-lipoxygenase expression, while exogenous LTB4 controls parasite growth. Infect Immun.

[35] Lemos HP, Grespan R, Vieira SM, Cunha TM, Verri WA, Fernandes KSS (2009). Prostaglandin mediates IL-23/IL-17-induced neutrophil migration by inhibiting IL-12 and IFN-gamma production. Proc Natl Acad Sci U S A.

[36] Evans HG, Roostalu U, Walter GJ, Gullick NJ, Frederiksen KS, Roberts CA (2014). TNF-alpha blockade induces IL-10 expression in human CD4+ T cells. Nat Commun.

[37] Abad Dar M, Hölscher C (2018). Arginase-1 is responsible for IL-13-mediated susceptibility to Trypanosoma cruzi infection. Front Immunol.

[38] Lee CG, Homer RJ, Zhu Z, Lanone S, Wang X, Koteliansky V (2001). Interleukin-13 induces tissue fibrosis by selectively stimulating and activating TGF-beta1. J Exp Med.

[39] Rochitte CE (2021). The importance of understanding the progression of myocardial fibrosis in chronic Chagas cardiomyopathy. Arq Bras Cardiol.

[40] Souza PE, Rocha MO, Rocha-Vieira E, Menezes CA, Chaves AC, Gollob KJ (2004). Monocytes from indeterminate and cardiac Chagas patients display distinct phenotypes associated with morbidity. Infect Immun.

[41] Gutierrez FR, Guedes PM, Gazzinelli RT, Silva JS (2009). The role of parasite persistence in the pathogenesis of Chagas heart disease. Parasite Immunol.

[42] Lim JY, Oh JH, Jung JR, Kim SM, Ryu CH, Kimet HT (2010). MK886-induced apoptosis depends on 5-LO expression level in human malignant glioma cells. J Neurooncol.

[43] Woo JS, Kim SM, Jeong CH, Ryu CH, Jeun SS (2013). Lipoxygenase inhibitor MK886 potentiates TRAIL-induced apoptosis through CHOP- and p38 MAPK-mediated up-regulation of death receptor 5 in malignant glioma. Biochem Biophys Res Commun.

[44] Nadarajan K, Balaram P, Khoo BY (2016). MK886 inhibits the pioglitazone-induced anti-invasion of MDA-MB-231 cells: association with PPARalpha/gamma, FGF4 and 5-LOX. Cytotechnology.

[45] da Silva-Souza HA, Lira MN, Costa-Junior HM, da Cruz CM, Vasconcellos JS, Mendes AN (2014). Inhibitors of the 5-lipoxygenase arachidonic acid pathway induce ATP release and ATP-dependent organic cation transport in macrophages. Biochim Biophys Acta.

[46] Crema E, Oliveira RM, Werneck AM, Pastore R, Martins A, Silva AA (2006). Manometric evaluation of upper esophageal sphincter in patients with indeterminate form of Chagas' disease. Rev Soc Bras Med Trop.

[47] Ferreira RC, Ianni BM, Abel LC, Buck P, Mady C, Kalil J (2003). Increased plasma levels of tumor necrosis factor-alpha in asymptomatic/"indeterminate" and Chagas cardiomyopathy patients. Mem Inst Oswaldo Cruz.

[48] Ribeiro AL, Nunes MP, Teixeira MM, Rocha MO (2012). Diagnosis and management of Chagas disease and cardiomyopathy. Nat Rev Cardiol.

[49] Fan XM, Tu SP, Lam SK, Wang WP, Wu J, Wong WM (2004). Five-lipoxygenase-activating protein inhibitor MK-886 induces apoptosis in gastric cancer through upregulation of p27kip1 and Bax. J Gastroenterol Hepatol.

[50] Teixeira LK, Fonseca BPF, Vieira-de-Abreu A (2005). IFN-gamma production by CD8+ T cells depends on NFAT1 transcription factor and regulates Th differentiation. J Immunol.

[51] Peres CM, Paula L, Medeiros AI, Sorgi CA, Soares EG, Carlos D (2007). Inhibition of leukotriene biosynthesis abrogates the host control of Mycobacterium tuberculosis. Microbes Infect.

[52] Shen F, Li N, Gade P, Kalvakolanu DV, Weibley T, Doble B (2009). IL-17 receptor signaling inhibits C/EBPbeta by sequential phosphorylation of the regulatory 2 domain. Sci Signal.

[53] Serio KJ, Reddy KV, Bigby TD (2005). Lipopolysaccharide induces 5-lipoxygenase-activating protein gene expression in THP-1 cells via a NF-kappaB and C/EBP-mediated mechanism. Am J Physiol Cell Physiol.

[54] Moltke JV, O'Leary CE, Barrett NA, Kanaoka Y, Austen KF, Locksley RM (2017). Leukotrienes provide an NFAT-dependent signal that synergizes with IL-33 to activate ILC2s. J Exp Med.

[55] Provost V, Langlois A, Chouinard F, Rola-Pleszczynski M, Chakir J, Flamandet N (2012). Leukotriene D4 and interleukin-13 cooperate to increase the release of eotaxin-3 by airway epithelial cells. PLoS One.

[56] Aluri J, Cooper MA, Schuettpelz LG (2021). Toll-Like Receptor Signaling in the Establishment and Function of the Immune System. Cells.

[57] Broad A, Kirby JA, Jones DEJ (2007). Toll-like receptor interactions: tolerance of MyD88-dependent cytokines but enhancement of MyD88-independent interferon-beta production. Immunology.

[58] Hassan HM, Bagalagel A, Diri R, Noor A, Almasri D, Bannan DF (2025). Blocking leukotriene receptors improve experimentally induced gastric ulcers in rats by inhibiting inflammation and apoptosis. Int J Immunopathol Pharmacol.

